# Deep Ensemble Learning Based Objective Grading of Macular Edema by Extracting Clinically Significant Findings from Fused Retinal Imaging Modalities

**DOI:** 10.3390/s19132970

**Published:** 2019-07-05

**Authors:** Bilal Hassan, Taimur Hassan, Bo Li, Ramsha Ahmed, Omar Hassan

**Affiliations:** 1 School of Automation Science and Electrical Engineering, Beihang University (BUAA), Beijing 100191, China; 2 Department of Electrical Engineering, Bahria University (BU), Islamabad 44000, Pakistan; 3 School of Computer Science and Engineering, Beihang University (BUAA), Beijing 100191, China; 4 School of Computer and Communication Engineering, University of Science & Technology Beijing (USTB), Beijing 100083, China; 5 Department of Electrical and Computer Engineering, Sir Syed CASE Institute of Technology (SSCIT), Islamabad 44000, Pakistan

**Keywords:** biomedical image processing, image analysis, image classification, machine intelligence, machine vision, optical coherence tomography, fundus photography

## Abstract

Macular edema (ME) is a retinal condition in which central vision of a patient is affected. ME leads to accumulation of fluid in the surrounding macular region resulting in a swollen macula. Optical coherence tomography (OCT) and the fundus photography are the two widely used retinal examination techniques that can effectively detect ME. Many researchers have utilized retinal fundus and OCT imaging for detecting ME. However, to the best of our knowledge, no work is found in the literature that fuses the findings from both retinal imaging modalities for the effective and more reliable diagnosis of ME. In this paper, we proposed an automated framework for the classification of ME and healthy eyes using retinal fundus and OCT scans. The proposed framework is based on deep ensemble learning where the input fundus and OCT scans are recognized through the deep convolutional neural network (CNN) and are processed accordingly. The processed scans are further passed to the second layer of the deep CNN model, which extracts the required feature descriptors from both images. The extracted descriptors are then concatenated together and are passed to the supervised hybrid classifier made through the ensemble of the artificial neural networks, support vector machines and naïve Bayes. The proposed framework has been trained on 73,791 retinal scans and is validated on 5100 scans of publicly available Zhang dataset and Rabbani dataset. The proposed framework achieved the accuracy of 94.33% for diagnosing ME and healthy subjects and achieved the mean dice coefficient of 0.9019 ± 0.04 for accurately extracting the retinal fluids, 0.7069 ± 0.11 for accurately extracting hard exudates and 0.8203 ± 0.03 for accurately extracting retinal blood vessels against the clinical markings.

## 1. Introduction

Visual impairments severely degrade the quality of life and have an adverse effect on people suffering from other chronic health issues. Currently, blindness is considered as a major health problem worldwide. According to the Global Burden of Disease (GBD) in their 2017 report (released on November 8th, 2018), loss of vision is categorized as the third leading form of impairments in humans and 48.2 million people are suffering from eye diseases all over the world. In addition to this, 39.6 million people have severe visual impairments whereas 279 million people and 969 million people have moderate to low visual impairments, respectively [1,2]. Moreover, most of the visual impairments that were reported are due to retinopathy.

The prime cause of retinopathy is diabetes mellitus (DM). DM is caused due to the destruction of pancreatic beta cells (β-cell) affecting the glucose metabolism of the candidate subject. DM is graded into two types. Type I DM specifies deficiency of insulin whereas Type II DM is associated with insulin resistance [3,4,5]. Apart from this, DM also affects other vital organs of the human body including eyes, kidney, heart, etc. [6]. Macula produces the central vision and it is the most critical part of retina. Any damage to macula results in the loss of central vision. The retinal diseases that affect the central vision of a person are collectively known as maculopathy. The most common form of maculopathy is ME, which is caused by the leakage of extracellular fluid from hyper-permeable capillaries in the macula of the retina. ME is clinically graded into different stages depending upon the affected area of macular thickening. However, early detection and laser photocoagulation can prevent sudden blindness in most of the cases. Moreover, many retinal complications are often treatable and according to the initiative of “VISION 2020: The Right to Sight”, different measures are being taken to eradicate avoidable blindness by the year 2020 [7]. At the same time, it is equally important to equip ophthalmologists with state-of-the-art retinal computer aided diagnostic systems for efficient detection and grading of retinopathy.

**Figure 1:**
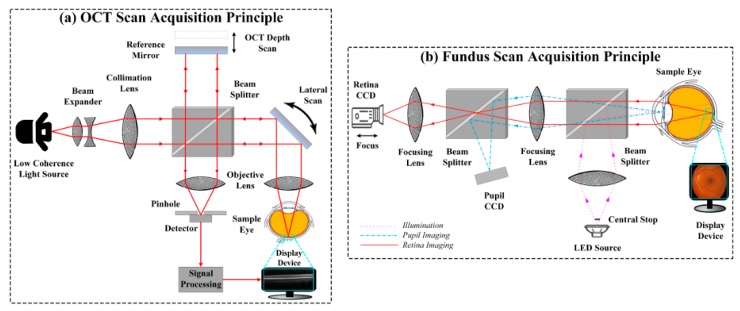
Working principle of retinal imaging modalities. (**a**) Optical coherence tomography (OCT) scan acquisition principle and (**b**) the fundus scan acquisition principle.

The two non-invasive imaging modalities that are clinically in practice for retinal examination, are OCT and fundus imagery [8]. OCT captures the tissue reflection through light coherency. For retinal examination, a beam is bombarded on the fundus of retina yielding a cross-sectional axial scan (A-scan) [9,10]. The A-scans are joined together to produce a brightness scan (B-scan). Since OCT captures the cross-sectional retina, so early progression of retinopathy can be easily visualized. The early identification of retinopathy effectively leads towards the better treatment. Retinal OCT imagery has revolutionized the clinical examination and eye treatment [11,12]. Figure 1a shows the basic OCT scan acquisition schematics, which is based on Michelson interferometer (MI). In MI, a monochromatic coherent light source is used to penetrate the human eye to produce a cross sectional retinal scan. Beam splitter at the center splits the light source into two separate beams where one beam is directed towards a reference mirror and the other travels to the subject’s eye. These two beams upon reflection get recombined into a single beam producing axial scan at the detector.

**Figure 2:**
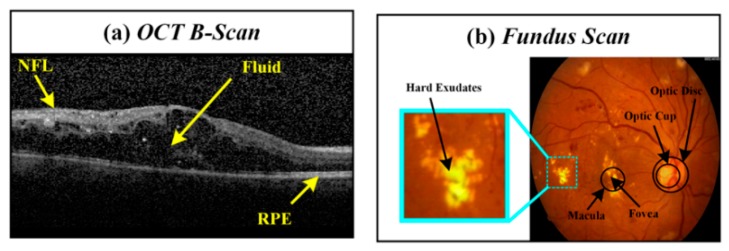
Appearance of macular edema symptoms in (**a**) an OCT B-scan and (**b**) fundus scan.

On the other hand, fundus photography also captures the central and peripheral retinal regions [13]. Figure 1b shows the acquisition principle of fundus imagery where a specialized microscope attached to a charge coupled device (CCD) camera for taking fundus photography. Fundus scans should ideally be taken in dim conditions. In certain circumstances, it becomes vital to consider all of the retinal examination techniques to fully analyze the pathological conditions of the human retina. The optical principle of the fundus camera is the same as of ophthalmoscopy, which acquires about two to five times enlarged inverted fundus scan [14,15]. The light passes through the series of biconvex lenses, which are used for focusing light to pass through the central aperture forming an annulus. After that, light passes through the cornea and falls on the fundus and hence the fundus scan appears on the display device, which can then be saved. The advantages of fundus photography are: it does not require pupil dilation, it is easy to use, it does not require a skilled user and it captures the images that can easily be examined by specialists at any time anywhere. However, apart from the high cost of equipment and non-portability, a major limitation of fundus photography is that it obtains a 2D representation of 3D semi-transparent retinal tissues projected onto the imaging plane, which is catered through OCT imagery. Figure 2 shows ME visualization in both OCT and fundus scans.

## 2. Related Work

In the past, many researchers have conducted clinical studies on analyzing ME using fundus and OCT scans [16,17,18] and concluded that OCT imaging provides better visualization of ME in comparison to fundus photography, especially in early stages where symptoms of ME are not relatively prominent. In addition to this, many studies have been conducted on devising automated algorithms for detecting ME from fundus or OCT scans individually. Most of the methods that use fundus images for the automated detection of ME are based on component segmentation, lesion detection and extraction of hard exudates (HE). Since in digital fundus scans, the contrast between HE and other retinal structures is relatively high, the most common approaches for detecting HE include marker-controlled watershed transformation [19], particle swarm optimization (PSO) based algorithm [20] and by means of local standard variation in a sliding window, morphological closing of the luminance channel and watershed transform [21]. However, illumination variations, which arise because of the changes in tissue pigmentation and imaging conditions, greatly affect these methods. Additionally, the methods based on extracting edge and color features are also proposed over the past for the segmentation of HE [22,23,24,25]. In general, such algorithms produce unsatisfactory results without including complex pre and post processing steps.

Different researchers have developed automated frameworks for the extraction of retinal layers and retinal fluids for analyzing ME affected pathologies [26,27,28,29]. Kernel regression and graph theory dynamic programming (KR + GTDP) [30] and software development life cycle (SDLC) [31] frameworks are also developed for segmenting retinal layers and retinal fluids in ME affected OCT scans. Srinivasan et al. [32] proposed a maculopathy detection framework using histogram of oriented gradients. Apart from this, deep learning frameworks [33,34,35] are also proposed recently for the automated extraction of retinal information from maculopathy affected OCT scans.

However, to the best of our knowledge, no method has been proposed in the past that fuses multiple retinal imaging modalities for objective evaluation of ME pathology. In this paper, we proposed a deep ensemble learning based framework that gives the objective grading ME pathology. The main contributions of our papers were as follows:

1. A novel method was presented in this paper that extracted the ME pathological symptoms from retinal fundus and OCT scans.
2. Instead of extracting handcrafted features, the proposed framework employed a deep convolutional neural network (CNN) model that gives the most relevant and useful features from retinal fundus and OCT scans for the objective evaluation of ME pathology irrespective of the scan acquisition machinery.
3. Many frameworks that have been proposed in the past were tested on a single dataset or on scans acquired through single OCT machinery. However, the proposed framework could give objective grading of ME pathology irrespective of OCT acquisition machinery and was rigorously tested on scans from different publicly available datasets.
4. The proposed framework employed an ensemble of artificial neural networks (ANN), support vector machines (SVM) and naïve Bayes (NB) for the in-depth grading of ME using both fundus and OCT retinal imaging modalities.
5. The proposed framework is adaptive and gives more weight to the clinical findings such as foveal swelling, fluid filled spaces and hard exudates while evaluating ME. This is achieved by fine-tuning the proposed CNN model on observing the critical ME symptoms from both fundus and OCT imagery.

Rest of the paper is organized as: Section 3 reports dataset details used in this study, Section 4 explains the proposed methodology, results are presented in Section 5 and Section 6 describes the detailed discussion about the proposed framework. Section 7 concludes the paper and highlights the future directions.

## 3. Datasets

The proposed framework has been tested on retinal fundus and OCT B-scans from multiple publicly available Rabbani and Zhang datasets. Zhang’s dataset only consisted of OCT scans of various retinal pathologies while Rabbani’s datasets had scans of fundus, fluorescein angiography (FA) and OCT retinal imaging modalities. We excluded the retinal pathologies other than healthy and ME in these datasets. The detailed description of the datasets that were used for training and evaluation purposes is listed in Table 1. All the scans within the datasets were marked by the expert clinicians and we used them as a ground truth in evaluating the performance of the proposed framework.

## 4. Proposed Methodology

**Figure 3:**
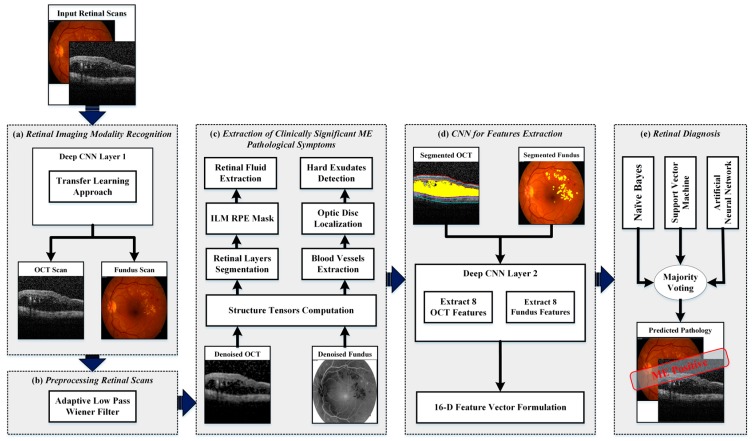
Proposed methodology for automated recognition and classification of healthy and macular edema (ME) affected eyes. (**a**) Retinal imaging modality recognition; (**b**) preprocessing retinal scans; (**c**) extraction of clinically significant ME pathological symptoms; (**d**) convolutional neural network (CNN) for feature extraction and (**e**) retinal diagnosis.

The proposed framework fuses retinal fundus and OCT imagery for the automated recognition and classification of ME and healthy subjects. The block diagram of the proposed framework is shown in Figure 3 where it can be observed that the proposed framework consisted of five major stages:

- Retinal imaging modality recognition;
- Preprocessing retinal scans;
- Extraction of clinically significant ME pathological symptoms;
- CNN for feature extraction;
- Retinal diagnosis.

At first, the input retinal scans were categorized as fundus or OCT through the first layer of the deep CNN model. Afterwards, different acquisition artifacts and unwanted noise content from both type of imagery were removed through the preprocessing stage. After enhancing the scans, the information about retinal layers, retinal fluids and the hard exudate regions were automatically extracted through the set of coherent tensors, which highlights the clinically significant pathological features of ME retinal syndrome. The extracted retinal information was then mapped on the original scan from which the distinct features were extracted through deep CNN models. The extracted features from both fundus and OCT imagery were concatenated together to form a feature vector upon which the candidate subject was graded. The detailed description of each stage is presented in the subsequent subsections below.

### 4.1. Retinal Imaging Modality Recognition

**Figure 4:**
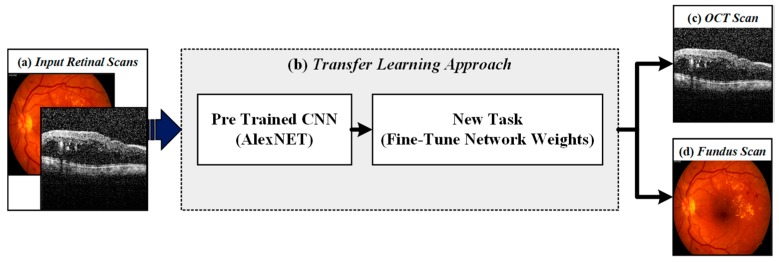
Retinal imaging modality recognition stage. (**a**) Input retinal scans; (**b**) transfer learning approach using AlexNet CNN architecture; (**c**) recognized OCT scan and (**d**) recognized fundus scan.

The first stage of the proposed framework was related to the automated recognition of retinal fundus and OCT scans. For this purpose, we utilized the pre-trained AlexNet model [42]. AlexNet is a 25-layered CNN architecture that is trained on an ImageNet dataset. We modified the classification layer of the AlexNet network and retrained it on the local image modality recognition training dataset through transfer learning. The transfer learning phase is shown in Figure 4 and the detailed description of the respective training dataset is presented in Table 1. The pretrained weights of the AlexNet model were very convergent for the recognition of retinal imaging modalities, which resulted in lesser training and fine-tuning time. The optimization during the training phase was performed through stochastic gradient descent (SGD) [43] where two 50% dropout layers were employed to reduce the overfitting. The main reason for employing AlexNet model instead of designing a CNN architecture from scratch is to achieve greater accuracy with the small amount of training dataset in a lesser time duration. Apart from this, the softmax function was used in the modified AlexNet architecture to compute final output probabilities. The softmax function is mathematically expressed in Equation (1) and the architectural description about AlexNet layers is presented in Table 2.

(1) $$\sigma {\left(X\right)}_{i}=\frac{e^{x_{i}}}{\sum_{j=1}^{N}e^{x_{j}}}$$

where, $X=\{x_{1},\;x_{2},\;\ldots \;x_{N}\}$ is the input vector. After each convolution layer, the rectified linear units (ReLU) layer is employed that ensures that only the positive values retain in the feature map (because the negative values reflect the changes, which are dissimilar within the input and the convolutional kernel). After the ReLU layer, the max pooling layer has been added, which only keeps the maximum values within the neighborhood, which ultimately shrinks the resultant feature map.

### 4.2. Preprocessing Retinal Scans

**Figure 5:**
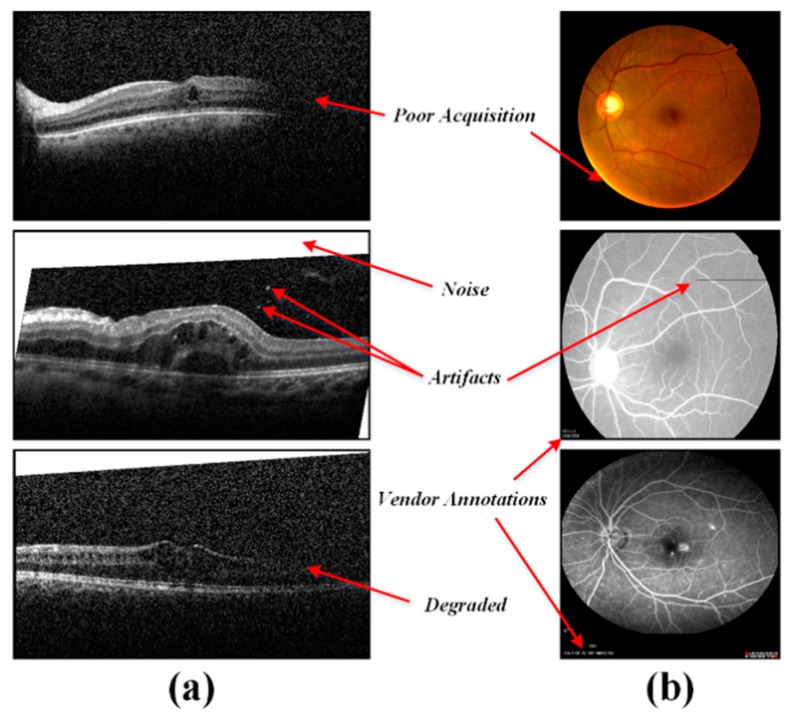
Degraded retinal scans. (**a**) OCT Scans and (**b**) fundus/ fluorescein angiography (FA) scans.

The acquisition of retinal scans is highly sensitive to the subject’s head and eye movements and this often leads towards the scan degradation. Apart from this, the acquisition machines add different kind of scan annotations, which greatly affects the automated retinal analysis. In order to cater such noisy artifacts, a preprocessing stage was added, which removes the noisy contents effectively, while enhancing the retina. Since the annotations are mostly added in the top and bottom rows of the respective B-scan. They are automatically removed by setting the first and last fifty rows to zero. This threshold was empirically selected by analyzing the scans within all the datasets. Apart from this, the degraded scan areas as shown in Figure 5 were automatically removed by searching for the first and last highly sharp transitions for each column within the respective scan and then by setting the values in the identified noisy regions with the mean of background pixels.

The preprocessing stage further enhances retinal portions by increasing their variability with the background and also by removing the noisy outliers. This is accomplished through an adaptive low pass Wiener filter, which uses a localized neighborhood of a candidate pixel for denoising [34]. The response of the Wiener filter is expressed in Equations (2)–(4):

(2) $$𝓂=\frac{1}{w_{h}w_{v}}\sum_{x_{i}\in w_{h}}\sum_{y_{i}\in w_{v}}O\left(x_{i},y_{i}\right),$$

(3) $${𝓈}^{2}=\frac{1}{w_{h}w_{v}}\sum_{x_{i}\in w_{h}}\sum_{y_{i}\in w_{v}}O^{2}\left(x_{i},y_{i}\right)-{𝓂}^{2},$$

(4) $$D\left(x_{i},y_{i}\right)=𝓂+\frac{{𝓈}^{2}+a^{2}}{{𝓈}^{2}}\left(O\left(x_{i},y_{i}\right)-𝓂\right),$$

where, $O\left(x_{i},y_{i}\right)$ and $D\left(x_{i},y_{i}\right)$ represent the pixels of the original and denoised retinal scan respectively, $w_{h}$ is the horizontal axis of denoising window while $w_{y}$ is the vertical axis of denoising window. Local estimated mean and variance are represented by 𝓂 and ${𝓈}^{2}$ respectively and ${𝒶}^{2}$ is the average of all estimated mean values.

### 4.3. Extraction of Clinically Significant ME Pathological Symptoms

ME is clinically graded into different categories as defined by the Early Treatment Diabetic Retinopathy Study (EDTRS). ME due to the presence of hard exudates and retinal fluids within the foveal diameter of 500 micrometers was considered to be clinically significant. ME outside this region was considered as non-clinically significant. Therefore, the accurate extraction of hard exudates, retinal fluid regions and the localization of fovea were very critical for effectively grading ME. Retinal fluids could be accurately observed through OCT scans while hard exudates were effectively visualized through fundus images. Therefore, the proposed framework, rather than relying on either fundus or OCT imagery, used both of them to effectively extract the retinal information for the reliable and objective grading of ME. In order to localize fovea, the proposed framework extracted the retinal layers from the OCT volume and measured the deepest inner limiting membrane (ILM) point within the foveal B-scan.

**Figure 6:**
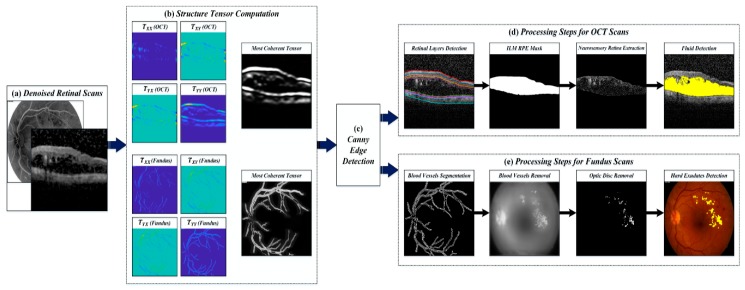
Extraction of clinically significant ME pathological symptoms. (**a**) Denoised retinal scans; (**b**) structure tensors computation stage; (**c**) detection of edges using the canny edge detection method; (**d**) processing steps for OCT scans and (**e**) processing steps for fundus scans.

The extraction of retinal information from both types of imagery was performed through structure coherence matrix, also known as structure tensor. Structure tensor has gained tremendous popularity in medical image processing because it provides low-level feature analysis and it is very useful for detecting corners, edges and boundaries [44]. Structure tensor also known as Förstner interest operator, is a second moment matrix which computes the gradients of an image by using Gaussian derivative filters as expressed in Equations (5)–(8):

(5) $$𝓈𝒯=\left[\begin{array}{cc} T_{𝓍𝓍}{}^{2} & T_{𝓍𝓎}\\ T_{𝓎𝓍} & T_{𝓎𝓎}{}^{2} \end{array}\right],$$

(6) $$T_{𝓍𝓍}{}^{2}=\sum_{x_{i}\in g_{x}}\sum_{y_{i}\in g_{y}}g\left(x_{i},\;y_{j}\right){\left[\varphi_{𝓍}D\left(x-x_{i},y-y_{j}\right)\right]}^{2},$$

(7) $$T_{𝓎𝓎}{}^{2}=\sum_{x_{i}\in g_{x}}\sum_{y_{i}\in g_{y}}g\left(x_{i},y_{j}\right){\left[\varphi_{𝓎}D\left(x-x_{i},y-y_{j}\right)\right]}^{2},$$

(8) $$T_{𝓍𝓎}=T_{𝓎𝓍}=\sum_{x_{i}\in g_{x}}\sum_{y_{i}\in g_{y}}g\left(x_{i,\;}y_{j}\right)[\varphi_{𝓍𝓎}D\left(x-x_{i},y-y_{j}\right)],$$

where, 𝓈T is the second order structure tensor matrix. $T_{𝓍𝓍}{}^{2}$ is the horizontally computed tensor, $T_{𝓎𝓎}{}^{2}$ is the vertically computed tensor and $T_{𝓍𝓎}$, $T_{𝓎𝓍}$ are the horizontal and vertical oriented tensors. $\varphi_{𝓍}$, $\varphi_{𝓎}$ and $\varphi_{𝓍𝓎}$ are the partial derivate of denoised image within the pixel neighborhood with respect to x, y and both x, y orientations. 𝑔(x,y) is the Gaussian window and D(x,y) is the de-noised retinal scan. Figure 6 shows the structure tensor computational stage. Structure tensor uses a set of eigenvalues to measure the degree of coherency and the tensor with maximum coherency is automatically selected for extracting retinal information [34].

After preprocessing the retinal fundus and OCT scans, the second moment matrix was automatically computed by the proposed framework for further analysis. 𝓈T from the retinal fundus scan was computed for the extraction of blood vascular patterns. Afterwards, the optic disc region was automatically localized by analyzing the high intensity retinal regions. The extraction of blood vessels and localization of optic disc region was performed in order to improve the segmentation of hard exudates regions. Since blood vessels contain high frequency components, so, the tensors present their detailed visualization while suppressing all other contents as evident from Figure 6b. After computing 𝓈T of the candidate fundus scan, the four coherent tensors were obtained. The best tensor ($T_{MAX}$) was then obtained by fusing $T_{XX}$ and $T_{YY}$ tensors, which together contained gives the maximum information about the blood vessels. Blood vessel segmentation in the proposed framework is quite robust as it can easily extract small blood capillaries as well, which are not even visible to the naked eye as shown in Figure 6e.

Structure coherence matrix of the retinal OCT scans is computed for the extraction of up to nine retinal layers [34]. Since most of the retinal layers are horizontally oriented so $T_{YY}$ will the most coherent tensor in 𝓈T for extracting layers information as evident from Figure 6b. After extracting the nine retinal layers, ILM and the retinal pigment epithelium (RPE) layers were used to generate a retinal mask, which was then multiplied by the candidate OCT B-scan for the extraction of retinal fluids [34]. The extraction retinal information was then overlaid onto the respective scan for the extraction of clinically significant feature set by the proposed CNN model.

### 4.4. CNN for Feature Extraction

**Figure 7:**
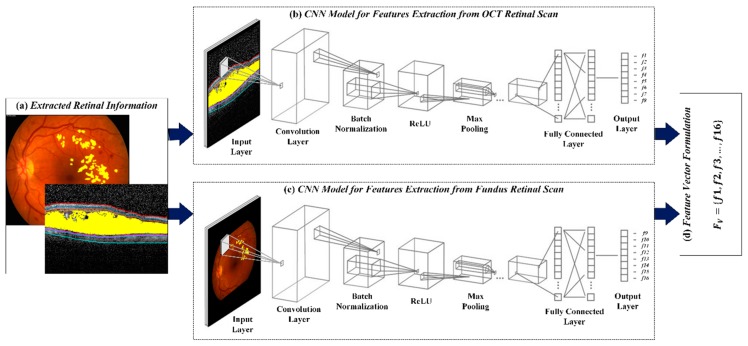
CNN for feature extraction. (**a**) Overlaid retinal scans with ME pathological symptoms; (**b**) CNN model for feature extraction from OCT retinal scans; (**c**) CNN model for feature extraction from retinal fundus scans and (**d**) 16-D feature vector containing eight OCT ($f_{1},f_{2},\ldots ,f_{8}$) and eight fundus features ($f_{9},f_{10},\ldots ,f_{16}$).

After extracting the hard exudates, retinal layers and retinal fluids, they were marked on the respective fundus and OCT scans for computing the distinct features to discriminate between healthy and ME affected subjects. These features were extracted through proposed CNN architecture. We designed a 14 layered structure tensor influenced CNN architecture containing one input layer, three convolution layers, three batch normalization layers, three ReLUs, two max pooling layers, one dropout layer with 50% threshold and one fully connected layer. The kernels within the convolution layers of the proposed CNN architecture contained weights that retain the structure tensor-based features while suppressing other content. This gave the significant variability between ME and healthy subjects. The proposed CNN model for feature extraction was designed from scratch and was trained on more than 0.07 million scans where the optimization was performed through SGD. In the proposed CNN model, the negative convolution sum values were removed through ReLU and the max pooling layer shrank the feature map to avoid unnecessary calculations. Since retinal fundus and OCT scans showed different clinically significant ME findings, therefore, the proposed CNN architecture extracted distinct features from both imaging modalities (i.e., it extracted eight distinct features from retinal fundus scan and eight distinct features from OCT images), which were then concatenated together to generate a 16-D feature vector. These sixteen features were then used to grade healthy and ME subjects. The proposed CNN model shows promising results of feature extraction after getting trained on the dataset mentioned in Table 1. This was due to the robust extraction of retinal information, which were mapped on the retinal scans from which proposed that the CNN model generates the most meaningful and distinctive features as shown in Figure 7. The detailed configuration of the proposed CNN model for feature extraction is presented Table 3, while Table 4 contains the sixteen extracted features from some of the healthy and ME affected scans. Figure 7 shows detailed CNN model for features extraction from both imaging modalities.

### 4.5. Retinal Diagnosis

After extracting the sixteen clinically significant features from retinal fundus and OCT imagery, they were concatenated together and were utilized by the hybrid classification system for grading ME. The hybrid classification model in the proposed framework consisted of an ensemble of ANN, SVM and NB. The final decision was computed by measuring the majority votes of all three classification models. The description of each classification model is presented below.

#### 4.5.1. Artificial Neural Networks

**Figure 8:**
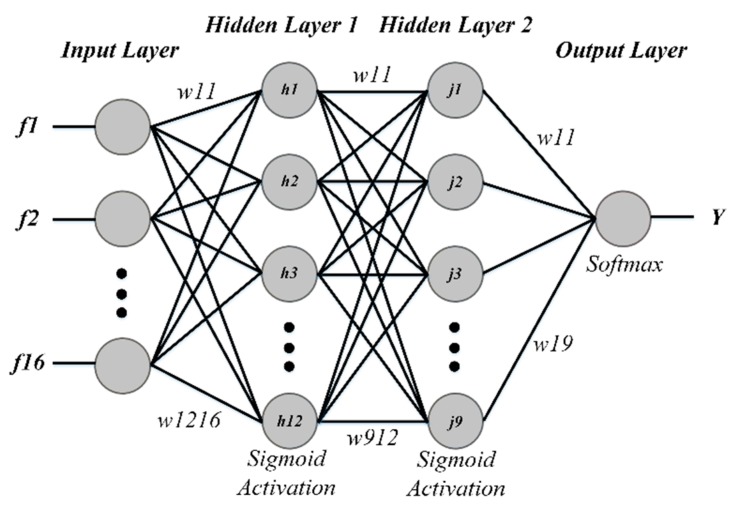
Architecture of an ANN model.

In this study, we used a feed forward artificial neural network classifier with one input layer, one output layer and two hidden layers. The input layer consisted of 16 nodes as per the extracted features. For hidden layers, we experimented with two to 40 nodes to find the optimum architecture (12 for the 1st hidden layer and nine for the 2nd hidden layer) of the neural network. A single output layer node gave the final classification probability. The sigmoid function was used for activation in each hidden layer whereas the final output layer contained softmax as the activation function. The weights during training were updated through gradient descent. Figure 8 shows the architecture of ANN used in the proposed study.

#### 4.5.2. Support Vector Machines

We used a SVM classifier as well in the proposed classification model. SVM is among the most extensively used classifier [34], and in this research a non-linear decision boundary was computed through Gaussian radial basis function (RBF) and multilayer perceptron (MLP) hyperplanes for predicting ME and healthy subjects based on the extracted feature vector ($F_{V}$).

#### 4.5.3. Naïve Bayes 

NB is a probabilistic classifier, which makes a decision based on the maximum a posteriori (MAP) rule. In this study, we used the NB classifier to determine the probability of ME and healthy classes through a 16-D feature vector. The category with the maximum probability was then automatically chosen as a diagnosis for the respective feature vector. The probabilities were computed through Bayes Rule as expressed in Equations (9) and (10):

(9) $$P\left(c_{i}|F_{v}\right)=P\left(F_{v}|c_{i}\right)P\left(c_{i}\right)/P\left(F_{v}\right),$$

(10) $$Y=arg_{c_{i}}\max\left[P\left(c_{i}|F_{v}\right)\right],$$

where, $c_{i}$ represents the healthy and ME class, $F_{v}$ is the 16-D test feature vector formulated during the feature extraction stage and Y represents the class assigned to the unlabeled scan, which has the largest probability given the $F_{v}$. $F_{v}$ contains eight distinct features from the retinal fundus scan and eight distinct features from the OCT scan. We used Gaussian distribution to calculate the likelihood $P\left(F_{v}|c_{i}\right)$.

**Figure 9:**
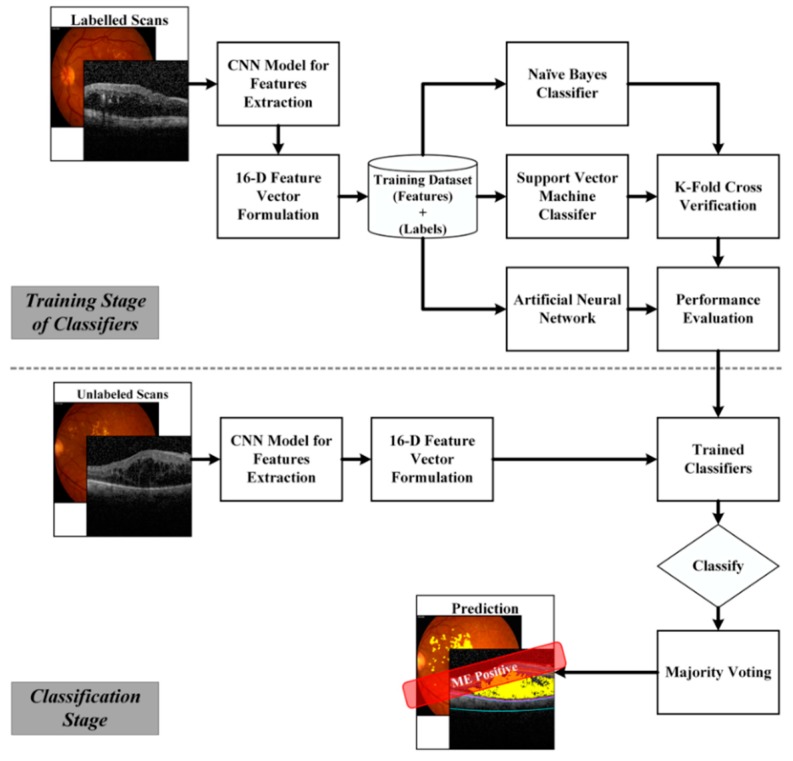
Block diagram of a hybrid classifier training and classification.

The detailed block diagram of classifiers training stage is shown in Figure 9. We used around 0.07 million retinal scans for training the hybrid classifier. Details of the training dataset are mentioned in Table 1. At first, sixteen distinct features were extracted from the labeled training scans to form a 16-D feature vector, which was then passed to all three classifiers separately and their decisions were finalized through majority voting. The performance of the proposed hybrid classifier during training was measured through K-fold cross validation as shown in Table 5 for different values of k. Once the classifiers achieved the desirable accuracy, they were used for retinal diagnosis of unlabeled scans during the classification stage as shown in Figure 9. Algorithm 1 summarizes the working flow of our proposed framework.


**Algorithm 1:** Proposed Framework

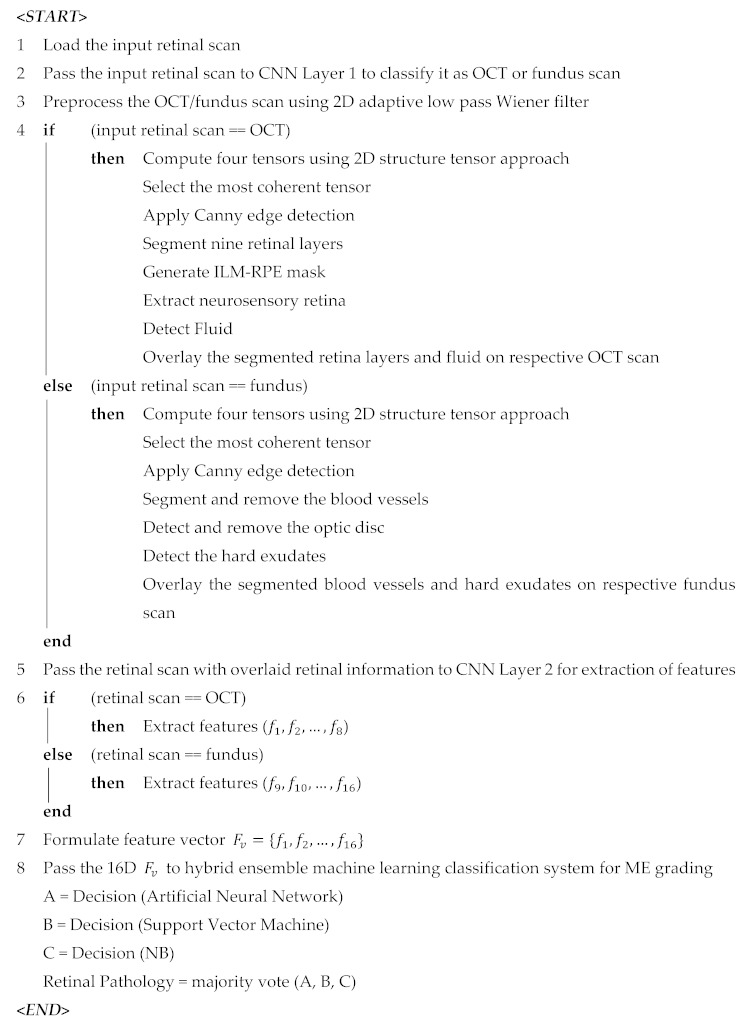



## 5. Results

We tested the proposed framework on an unlabeled dataset consisting of 5000 OCT B-scans out of which 2500 were of ME affected eyes and 2500 were of healthy eyes and 100 fundus scans with the same ratio of ME and healthy eyes. Since the feature vector is generated by concatenating the extracted features from both fundus and OCT scans, therefore, we individually computed the mean dice coefficient for measuring the performance of extracting hard exudates, blood vessels and retinal fluids.

**Figure 10:**
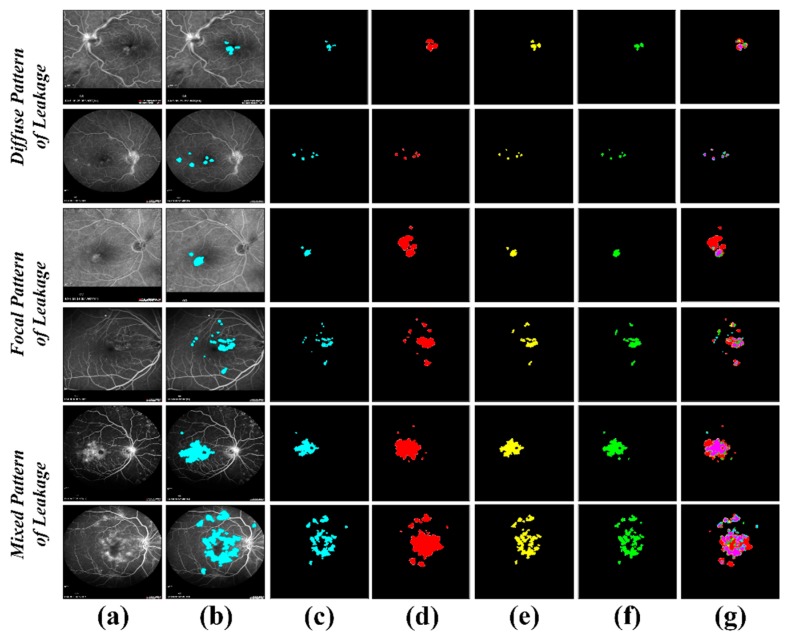
Segmented hard exudates region in the [37] dataset. (**a**) Original FA scans; (**b**) overlaid hard exudates region on original FA scans; (**c**) extracted hard exudates region through the proposed system; (**d**) Grader 1 markings of hard exudates region; (**e**) Grader 2 markings of hard exudates region; (**f**) Grader 3 markings of hard exudates region; (**g**) hard exudates extraction against expert markings—red, yellow and green represents the expert markings of Grader 1, 2 and 3, respectively, cyan represents the hard exudates region extracted through the proposed system and magenta represents the overlapped region of hard exudates.

The dataset in [37] consisted of 24 diabetic macular edema eyes with seven diffuse pattern of fluid leakage, 10 focal pattern of leakage and seven mixed pattern of leakage. They also provided three different expert markings of hard exudates for all these cases, which we used in validating the performance of the proposed system in extracting hard exudates regions as shown in Table 6. It can be observed from Table 6 that the proposed framework achieved the overall mean dice coefficient of 0.7069 ± 0.11 in extracting hard exudates. Figure 10 shows the visual comparison of the proposed framework for extracting hard exudates with three different expert markings.

**Figure 11:**
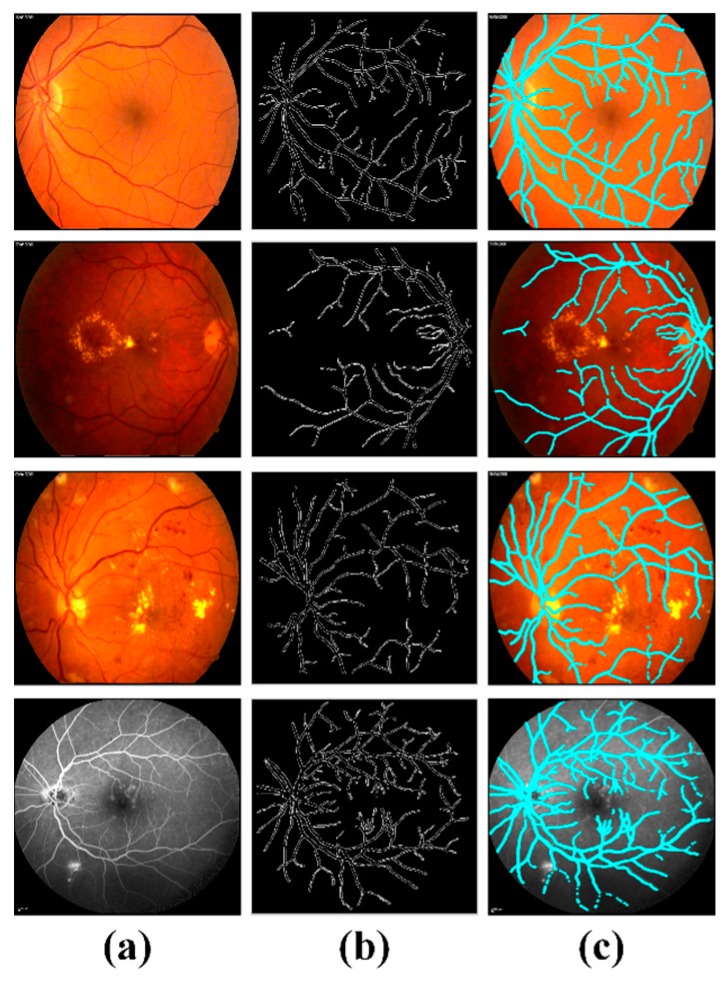
Extracted blood vessels through the proposed system in the [37,41] datasets. (**a**) Original fundus/FA scans; (**b**) extracted blood vessels through proposed system and (**c**) overlaid blood vessels on original fundus/FA scans.

Since the annotations against blood vessels in fundus/FA scans and retinal fluids in OCT scans were not available in the datasets used in this study, we arranged these annotations through a local expert clinician for comparative analysis. We evaluated the efficiency of the proposed system for blood vessels extraction through mean dice coefficient computed against the manual markings done by a local clinician as shown in Table 7. We obtained the overall mean dice coefficient of 0.8589 ± 0.04 for blood vessels segmentation in the case of healthy eyes and 0.8012 ± 0.03 in the case of ME affected eyes. Whereas, for both retinal conditions we achieved the overall mean dice coefficient of 0.8203 ± 0.03. These results validate the accuracy of proposed systems in blood vessels segmentation against various retinal pathologies, even in the presence of hard exudates, hemorrhages and micro-aneurysms in ME fundus/FA scans. It shows the effectiveness of the proposed method in detailed extraction of blood vessels. Figure 11 shows the extracted blood vessels by the proposed system in healthy and ME affected fundus/FA scans.

Similarly, we evaluated the performance of the proposed system for the extraction of retinal fluids through mean dice coefficient computed against the manual markings done by a local clinician as shown in Table 8. We obtained the overall mean dice coefficient of 0.9026 ± 0.03 for retinal fluid extraction on the Rabbani dataset [36] and 0.9012 ± 0.04 on the Zhang dataset [35].

**Figure 12:**
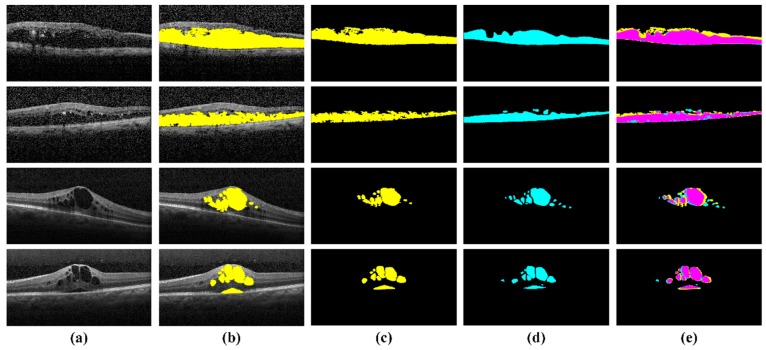
Extracted retinal fluid regions in the [35,36] datasets. (**a**) Original OCT scans; (**b**) overlaid retinal fluid regions on original OCT scans; (**c**) extracted retinal fluid regions through the proposed system; (**d**) local clinician markings of retinal fluid regions (**e**) retinal fluid extraction against expert markings—cyan represents the expert markings of local clinician, yellow represents the retinal fluid regions extracted through the proposed system and magenta represents the overlapped region of retinal fluids.

Whereas, for both the datasets we achieved the overall mean dice coefficient of 0.9019 ± 0.04. These results show that the proposed method performed well in retinal fluid extraction irrespective of the datasets and the OCT acquisition equipment. Figure 12 shows the extracted retinal fluid by the proposed system in healthy and ME affected OCT scans.

Moreover, we performed a classification of healthy and ME scans based on a 16-D feature vector extracted through the proposed CNN model. We passed the 16-D feature vector extracted from retinal fundus and OCT imagery to the proposed hybrid classifier for grading ME. The hybrid classification model in the proposed framework consisted of an ensemble of ANN, SVM and NB. The final decision was computed by measuring the majority votes of all three classification models. The hybrid classifier correctly classified 94.33% of all the unlabeled dataset scans, while individual performances of each classifier along with other methods reported in literature are listed in Table 9. We used sensitivity (SE), specificity (SP), positive predictive values (PPV), negative predictive values (NPV) and diagnostic accuracy (A) as the five measuring metrics to evaluate the hybrid classifier as expressed in Equations (11)–(15):

(11) $$Sensitivity=\;\frac{T_{P}}{T_{P}+F_{N}},$$

(12) $$Specificity=\;\frac{T_{N}}{T_{N}+F_{P}},$$

(13) $$PPV=\;\frac{T_{P}}{T_{P}+F_{P}},$$

(14) $$NPV=\;\frac{T_{N}}{T_{N}+F_{N}},$$

(15) $$Diagnostic\;Accuracy=\;\frac{T_{P}+\;T_{N}}{T_{P}+T_{N}+F_{P}+F_{N}}.$$

$T_{P}$ and $T_{N}$ are the true positives and true negatives respectively, which specify the correctly classified (CC) cases. In this study, $T_{P}$ indicates whether the input scan was macular edema and it was classified as macular edema too, while $T_{N}$ represents the cases where actual input scan was of healthy eye and the classification also showed it as healthy. $F_{P}$ and $F_{N}$ stands for false positive and false negative, respectively, these are false classification indicators. $F_{P}$ cases are those in which actual input scan was of healthy eye and classifier classified it as ME, while $F_{N}$ is the reverse of $F_{P}$.

**Figure 13:**
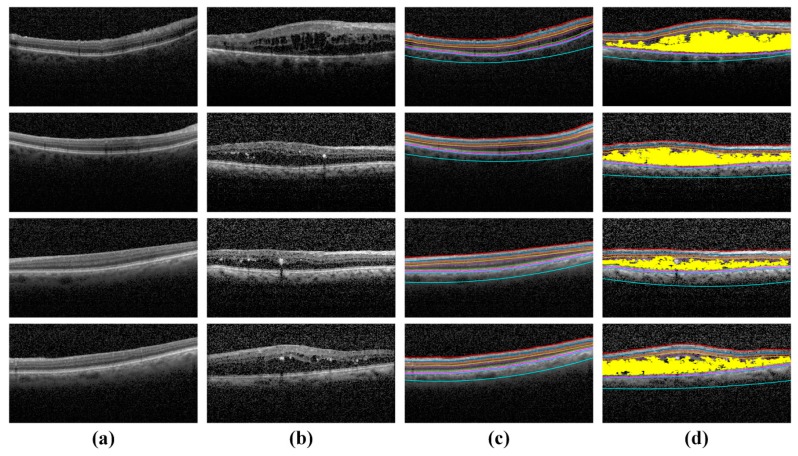
Healthy and ME OCT scans in the Rabbani dataset [36], which are processed by the proposed system. (**a**) Original healthy OCT B-scans; (**b**) original ME OCT B-scans; (**c**) classified as healthy by the proposed system and (**d**) classified as ME by the proposed system.

**Figure 14:**
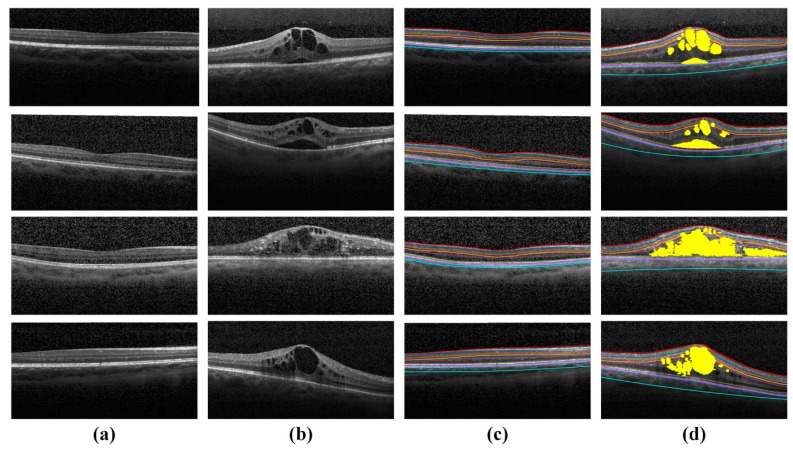
Healthy and ME OCT scans in the Zhang dataset [35], which are processed by the proposed system. (**a**) Original healthy OCT B-scans; (**b**) original ME OCT B-scans; (**c**) classified as healthy by the proposed system and (**d**) classified as ME by the proposed system.

**Figure 15:**
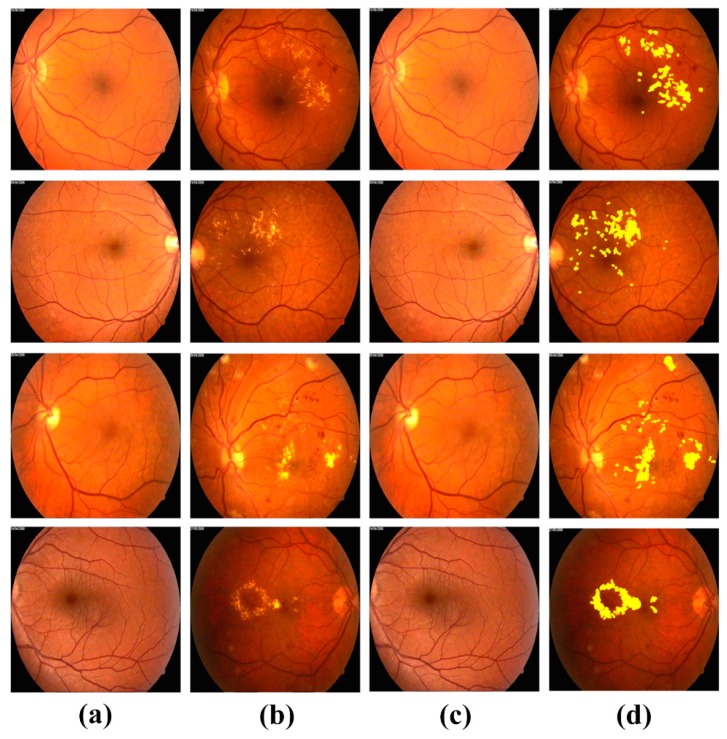
Healthy and ME fundus scans in the Rabbani Dataset [39,40], which are processed by the proposed system. (**a**) Original healthy fundus scans; (**b**) original ME fundus scans; (**c**) classified as healthy by the proposed system and (**d**) classified as ME by the proposed system.

Figure 13 and Figure 14 shows some healthy and ME OCT cases from both Rabbani [36] and Zhang [35] datasets, which are correctly processed by the proposed framework whereas Figure 15 shows some of the healthy and ME fundus scans, which were correctly processed by the proposed system.

**Figure 16:**
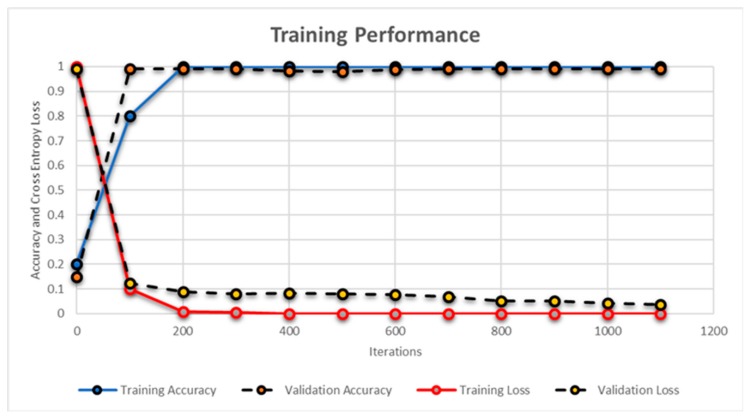
Training performance of the AlexNet model. Blue line shows the training accuracy, red line shows the training loss and the dashed black lines shows the validation accuracy and validation loss.

**Figure 17:**
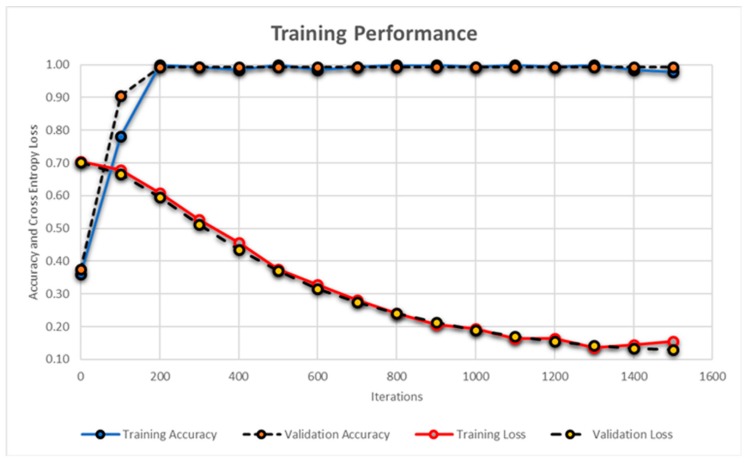
Training performance of the proposed CNN model. Blue line shows the training accuracy, red line shows the training loss and the dashed black lines shows the validation accuracy and validation loss.

Figure 16 and Figure 17 shows the training performance of AlexNet and the proposed CNN model for the modality recognition and feature extraction, respectively. The training of AlexNet was conducted for 40 epochs where each epoch was completed in 35 iterations. The training of the proposed CNN model for feature extraction was conducted for 30 epochs where each epoch contained 50 iterations. The performance of the AlexNet and proposed CNN model during training phase was measured through the accuracy and cross-entropy loss function as expressed in Equation (16):

(16) $$C_{L}=\Sigma I_{F_{V},w}log\left(P_{F_{V},w}\right)$$

where $I_{F_{V},w}$ is an indicator that the w is the correct class for the feature vector $F_{V}$, $P_{F_{V},w}$ is the probability computed for $F_{V}$ that it belongs to class w and $C_{L}$ is the cross-entropy loss. The summation in Equation (9) runs for the total number of classes.

Apart from this, for every 100 iterations, the validation was performed where validation performance was also measured through accuracy and cross-entropy loss function. The validation was performed in order to get the unbiased evaluation of the candidate model during the training phase as evident from Figure 16 and Figure 17. Furthermore, we employed 50% dropout layers within each model to reduce overfitting on the dataset. The proposed CNN model achieved the accuracy of 99.23% in 1500 iterations during the training phase while the AlexNet model achieved the accuracy of 98.79%. These results were obtained through MATLAB R2018a and Table 10 shows the details of systems and software along with the average time required for computing the results by each classifier. Although, the average time of hybrid classifier was a few seconds more than individual classifiers, the accuracy achieved by the proposed classification model was 94.33%.

## 6. Discussion

A deep retinal diagnostic framework was proposed here that combines retinal fundus and OCT imagery for the extraction of clinically significant ME findings and uses the extracted information for the reliable and accurate grading of ME. According to EDTRS, ME was clinically graded based upon the locality of edema with respect to fovea i.e., if the retinal fluids or hard exudates are observed within the foveal diameter of 500 micrometers, then ME is graded as clinically significant otherwise it is graded as non-clinically significant. Clinically significant macular edema is more critical as compared to non-clinically significant macular edema as it produces retinal thickening near the fovea, which causes non-recoverable visual impairments (or even blindness). Retinal fundus and OCT imagery are the most common and non-invasive retinal examination techniques, which depicts the prominent symptoms of retinopathy. OCT imagery shows the early symptoms of retinopathy due to its ability to present retinal cross-sectional regions. Therefore, the retinal blood vessels leakages and retinal fluids accumulation can be easily visualized through OCT scans. However, accurate visualization of hard exudates from OCT imagery is a very cumbersome task, therefore, the retinal fundus scans are clinically used for this purpose. To the best of our knowledge, all the retinal diagnostic frameworks that have been proposed in the past for ME diagnosis are based on single retinal imaging modality, which do not completely depict the retinal abnormalities. The proposed framework is unique as it fuses the findings from both retinal fundus and OCT imagery for the effective, reliable and objective diagnosis as well as grading of ME subjects (especially those having a diabetic history). The proposed framework works in a way that it first recognizes the type of imagery through the pre-trained AlexNet CNN model. The retinal imaging modality recognition is one of the crucial steps of the proposed framework since both images do not contain any metadata that can depict their unique information or description. Therefore, in order to develop a generalized framework that can perform automated analysis and can automatically mass screen retinal patients, the respective imagery has to be automatically recognized first. After recognizing the retinal images, the proposed framework extracts the retinal layers and retinal fluids from the candidate OCT scans and it also extracts the hard exudate regions from the fundus scans. The extracted retinal information is then overlaid onto the respective scans and the annotated scans are then passed to the proposed CNN model, which extracts the eight distinct features from the annotated fundus scan and eight distinct features from the annotated fundus scans. These features are fused together to form a 16-D feature vector, which is passed to the proposed hybrid classifier formed through the ensemble of ANN, SVM and NB. One of the major aims of the proposed framework was to accurately diagnose and grade ME pathologies. Since ME is clinically graded into different categories depending upon the disease severity levels so in order to get reliable and accurate diagnosis, the hybrid classification was proposed that gives a decision based upon the majority votes obtained through all the three supervised classifiers. This increases the diagnostic performance of the proposed framework without compromising the time performance as evident from Table 10. Apart from this, the proposed framework was extensively tested on multiple publicly available datasets and was compared with state-of-the-art solutions against different metrics and ground truths (provided by expert clinicians) as evident from the results section. Table 9 depicts the detailed diagnostic comparison with other existing solutions where it can be seen that the proposed framework was the only generic framework that was validated on multiple publicly available datasets containing both retinal fundus and OCT imagery and achieved the diagnostic accuracy of 94.33%.

## 7. Conclusions and Future Work

In this paper, we proposed a computer aided diagnostics method for segmentation of retinal pathological symptoms and classification of macular edema using two retinal imaging modalities (OCT and fundus imaging). The proposed framework was based on a hybrid classification model in which 16 unique features are extracted for distinguishing macular edema cases from healthy ones. The dataset used for conducting this study consisted of more than 78,891 retinal scans in total, out of which we used 73,791 scans for training purpose and 5100 for evaluation purpose. The proposed classification model correctly classified 4811 retinal scans, achieving 94.33% accuracy. The proposed system was quite robust in general, insensitive to OCT B-scans orientations and performed extremely well against the noisy and degraded scans as shown in Figure 5. Moreover, the proposed technique could be optimized for detecting other ocular diseases such as age-related macular degeneration (ARMD), idiopathic central serous chorioretinopathy (CSCR), Glaucoma, diabetic retinopathy, etc., as well as for segmenting other retinal layers. It could also be extended for the 3D modeling of the human retina.

## Figures and Tables

**Table 1:** Details of the dataset used for training and testing purposes.

Dataset ^a^	Imaging Modality ^b^		Retinal Pathology ^b^		Scans Dimension(s)	
	OCT	Fundus/FA	Healthy	ME	OCT	Fundus/FA
1 [36]	2764	-	1628	1136	496 × 512	-
2 [37]	-	24	-	24	-	512 × 612
						768 × 868
3 [38]	12,800	100	12,900	-	512 × 650	1612 × 1536
4 [39]	-	120	60	60	-	720 × 576
5 [40]	-	35	-	35	-	720 × 576
6 [41]	-	60	25	35	-	720 × 576
7 [35]	62,988	-	51,390	11,598	512 × 496	-
					512 × 512	
					768 × 496	
					1024 × 496	
					1536 × 496	
Total	78,552	339	66,003	12,888	-	
**Split**	**Training**				**Validation**	
**Modality**	**OCT**	**Fundus/FA**			**OCT**	**Fundus/FA**
**Total Scans**	73,552	239			5000	100
**Healthy**	63,318	135			2500	50
**ME**	10,234	104			2500	50

^a^ We only considered OCT and fundus imaging modalities consisting of healthy and ME retinal pathologies from these datasets; ^b^ the count shows the total number of scans in these datasets (including all the B-scans in OCT volumes).

**Table 2:** Function wise details of the AlexNet architecture.

Function	Layer	Description
Input Image	1	227 × 227 × 3 images
Convolution	2	9611 × 11 × 3 convolutions
	6	2565 × 5 × 48 convolutions
	10	3843 × 3 × 256 convolutions
	12	3843 × 3 × 192 convolutions
	14	2563 × 3 × 192 convolutions
ReLU	3	Assigns ‘0’ to non-positive values
	7	
	11	
	13	
	15	
	18	
	21	
Max Pooling	5	3 × 3 max pooling
	9	
	16	
Dropout	19	50% dropout
	22	
Normalization	4	Five channels per element
	8	
Fully Connected	17	4096 fully connected layer
	20	
	23	
Softmax	24	Softmax activation function
Output	25	Two Classes (OCT, Fundus)

**Table 3:** Proposed structure tensor influenced CNN architecture.

1	Input layer	227 × 227 × 3 images with ‘zerocenter’ normalization
2	Convolution	89 × 9 × 3 convolutions with stride [1,1] and padding ‘same’
3	Batch Normalization	Batch normalization with eight channels
4	ReLU	Rectified Linear Units
5	Max Pooling	2 × 2 max pooling with stride [2,2] and padding [0,0,0,0]
6	Convolution	169 × 9 × 8 convolutions with stride [1,1] and padding ‘same’
7	Batch Normalization	Batch normalization with 16 channels
8	ReLU	Rectified Linear Units
9	Dropout	50% Dropout
10	Max Pooling	2 × 2 max pooling with stride [2,2] and padding [0,0,0,0]
11	Convolution	329 × 9 × 16 convolutions with stride [1,1] and padding ‘same’
12	Batch Normalization	Batch normalization with 32 channels
13	ReLU	Rectified Linear Units
14	Fully Connected	Fully connected layer giving the significant eight features

**Table 4:** Selected fundus and OCT features from healthy and ME subjects.

Features		Healthy				Macular Edema			
		Case 1	Case 2	Case 3	Mean ^a^	Case 1	Case 2	Case 3	Mean ^a^
**OCT**	F1	2.28	1.53	1.69	1.51	1.97	−0.79	−2.62	1.17
	F2	2.13	4.35	1.58	2.18	0.58	0.63	1.29	0.86
	F3	−3.13	−1.92	−6.4	−3.57	−0.85	2.73	1.73	−1.56
	F4	4.83	0.84	1.48	2.87	−0.29	1.3	−3.38	0.19
	F5	0.59	0.28	0.44	0.29	1.9	1.35	0.76	0.68
	F6	4.82	1.87	7.59	4.46	2.73	3.29	2.59	2.93
	F7	1.03	0.37	2	0.71	−0.07	0.64	0.19	0.26
	F8	−0.65	0.94	0.58	−0.69	1.61	1.34	1.4	1.38
**Fundus**	F9	−1.85	0.61	−1.62	−0.95	0.3	2.3	0.1	0.27
	F10	2.67	1.4	2.51	2.93	1.28	1.42	1.17	1.09
	F11	−1.77	−7.83	−1.7	−1.14	−2.54	−2.11	−3.06	−2.71
	F12	1.29	0.18	0.01	0.26	1.44	1.13	1.46	1.43
	F13	1.61	−0.09	0.66	0.25	0.02	−2.49	0.81	−0.11
	F14	2.37	1.65	2.44	0.81	2.75	4.61	2.24	3.12
	F15	1.7	0.14	0.68	0.72	−1.04	1.46	−0.26	0.07
	F16	1.26	0.82	1.25	1.17	0.8	0.62	0.98	0.57

^a^ Mean value was computed using all the scans in a validation dataset.

**Table 5:** Classifiers K-Fold cross validation performance.

K	Max Accuracy Achieved		
	ANN	SVM	NB
2	0.816	0.809	0.794
3	0.893	0.841	0.829
4	0.914	0.874	0.864
6	0.948	0.907	0.917
8	0.972	0.925	0.942
**10**	**0.991**	**0.966**	**0.980**
11	0.985	0.948	0.961
12	0.973	0.929	0.944

**Table 6:** Mean dice coefficient for hard exudates segmentation against the expert markings [37].

Leakage Pattern	Scans	Against Grader 1	Against Grader 2	Against Grader 3
Diffuse	1	0.7529	0.8931	0.8372
	2	0.3573	0.5973	0.6698
	3	0.6838	0.6727	0.6744
	4	0.6718	0.7391	0.7739
Focal	1	0.5871	0.6397	0.6928
	2	0.2339	0.7288	0.694
	3	0.7169	0.8275	0.8631
	4	0.3887	0.6349	0.634
Mixed	1	0.6035	0.882	0.875
	2	0.5941	0.6691	0.7329
	3	0.6551	0.7938	0.8339
	4	0.5582	0.7661	0.7851
**Mean ± STD** **(All Dataset)**		**0.5726 ± 0.16**	**0.7669 ± 0.10**	**0.7813 ± 0.08**
**Mean ± STD** **(Overall)**		**0.7069 ± 0.11**		

**Table 7:** Mean dice coefficient for blood vessels segmentation against a local clinician’s annotations.

Scans	Rabbani Dataset 1 [41]		Rabbani Dataset 2 [37]
	Healthy	ME	ME
1	0.7817	0.7871	0.7914
2	0.8808	0.8315	0.8135
3	0.8747	0.8197	0.7861
4	0.7983	0.7890	0.8047
5	0.8669	0.8444	0.7971
6	0.8518	0.8071	0.8168
7	0.8811	0.8004	0.7896
8	0.8190	0.7927	0.8238
9	0.8468	0.8058	0.8275
10	0.8617	0.7871	0.7914
**Mean ± STD** **(All Dataset)**	**0.8589 ± 0.04**	**0.8185 ± 0.03**	**0.7839 ± 0.02**
**Mean ± STD** **(Overall)**	**0.8203 ± 0.03**		

**Table 8:** Mean dice coefficient for retinal fluids extraction against a local clinician’s annotations.

Scans	Rabbani Dataset [36]	Zhang Dataset [35]
1	0.9194	0.9152
2	0.8689	0.8560
3	0.9082	0.9351
4	0.8551	0.9145
5	0.8726	0.9243
6	0.9322	0.8796
7	0.9238	0.8986
8	0.8887	0.8731
9	0.9162	0.8766
10	0.8724	0.9259
**Mean ± STD** **(All Dataset)**	**0.9026 ± 0.03**	**0.9012 ± 0.04**
**Mean ± STD** **(Overall)**	**0.9019 ± 0.04**	

**Table 9:** Measure outcomes of the proposed ensemble hybrid classifier in comparison to other state-of-the-art techniques.

Methods		Validation Dataset		CC		TP	TN	FP	FN	SE	SP	PPV	NPV	A
		OCT	Fundus	OCT	Fundus									
**Proposed**	**ANN**	**5000 ^R, Z^**	**100 ^R^**	**4653**	**94**	**2457**	**2291**	**259**	**93**	**0.96**	**0.90**	**0.90**	**0.96**	**0.93**
	**SVM**			**4648**	**93**	**2407**	**2322**	**228**	**143**	**0.94**	**0.91**	**0.91**	**0.94**	**0.92**
	**NB**			**4559**	**92**	**2374**	**2289**	**261**	**176**	**0.93**	**0.90**	**0.90**	**0.93**	**0.91**
	**Hybrid**			**4716**	**95**	**2473**	**2338**	**212**	**77**	**0.97**	**0.92**	**0.92**	**0.97**	**0.94**
[19]		✗	20 ^Ψ, ψ^	✗	-	-	-	-	-	0.943*	1*	0.92*	-	-
										0.967^^^	1^^^	0.949^^^	-	-
[20]		✗	100 ^ϕ^	✗	93	33	60	0	7	0.825	1	-	-	0.93
[21]		✗	30	✗	-	-	13	2	-	0.928	-	0.924	-	-
[23]		✗	150 ^ζ^	✗	-	72	71	-	-	0.96	0.946	-	-	-
[24]		✗	400 ^ϕ^	✗	-	-	-	-	-	0.95	0.9	-	-	-
			104 ^ξ^							1	0.74			
[25]		✗	15 ^ψ^	✗	-	-	-	-	-	0.978	0.99	0.833	-	-
			15 ^C^							0.907	0.994	0.74	-	-
[26]		30 ^D^	✗	28	✗	15	13	-	-	1	0.933	-	-	-
[27]		19 ^C^	✗	-	✗	-	-	-	-	0.91	0.96	-	-	-
[28]		16	✗	-	✗	-	-	-	-	-	-	-	-	0.875
[29]		90 ^B^	✗	88	✗	60	28	-	-	1	0.933			0.977
[31]		50 ^B^	✗	42	✗	28	14	-	-	0.93	0.8	-	-	0.84
[32]		45 ^D^	✗	43	✗	30	13	-	-	1	0.866	-	-	-
[34]		42281 ^D^	✗	-	✗	-	-	-	-	0.991	0.986	-	-	0.985
		4260 ^B^												
[35]		500 ^Z^	✗	483	✗	237	246	-	-	0.968	0.996	-	-	0.982

^Ψ^Digital Retinal Images for Vessel Extraction (DRIVE) Dataset, ^ψ^Structured Analysis of the Retina (STARE) Dataset, ^ϕ^Methods to Evaluate Segmentation and Indexing Techniques in the field of Retinal Ophthalmology (MESSIDOR) Dataset, ^ζ^Bristol Eye Hospital Dataset, ^ξ^Hamilton Eye Institute Macular Edema (HEI-DMED) Dataset, ^C^Custom Dataset used by the authors, ^D^DUKE Dataset, ^B^Biomedical Image and Signal Analysis (BIOMISA) Dataset, ^Z^Zhang Dataset, ^R^Rabbani Dataset, *Results with fixed threshold, ^Results with variable threshold; ANN = Artificial Neural Network, SVM = Support Vector Machine, NB = Naïve Bayes Classifier, CC = Correctly Classified, TP = True Positive, TN = True Negative, FP = False Positive, FN = False Negative, SE = Sensitivity, SP = Specificity, PPV = Positive Predicted Values, NPV = Negative Predicted Values and A = Diagnostic Accuracy.

**Table 10:** Details of the system and software used for conducting this research.

System		Software		Average Time for Single Classification (seconds)		
				Classifier	OCT	Fundus
**Made**	**DELL**	Windows 10 Pro 64-bit	MATLAB R2018a	ANN	4.6	3.3
Processor	i7-4500U @ 2.4GHz			SVM	5.7	4.2
RAM	8GB DDR2			NB	3.2	1.8
Graphics	AMD HD 8670M			Hybrid	6.8	5.1
